# Transposon Tagging of a Male-Sterility, Female-Sterility Gene, *St8*, Revealed that the Meiotic MER3 DNA Helicase Activity Is Essential for Fertility in Soybean

**DOI:** 10.1371/journal.pone.0150482

**Published:** 2016-03-01

**Authors:** Jordan Baumbach, Ramesh N. Pudake, Callie Johnson, Kaylin Kleinhans, Alexandrea Ollhoff, Reid G. Palmer, Madan K. Bhattacharyya, Devinder Sandhu

**Affiliations:** 1 Department of Biology, University of Wisconsin-Stevens Point, Stevens Point, Wiconsin, 54481, United States of America; 2 Department of Agronomy, Iowa State University, Ames, Iowa, 50011, United States of America; 3 USDA-ARS Salinity Lab., 450 W. Big Springs Rd., Riverside, California, 92507, United States of America; McGill University, CANADA

## Abstract

The *W4* locus in soybean encodes a dihydroflavonol-4-reductase (DFR2) that regulates pigmentation patterns in flowers and hypocotyl. The mutable *w4-m* allele that governs variegated flowers has arisen through insertion of a CACTA-type transposable element, *Tgm9*, in *DFR2*. In the *w4-m* line, reversion from variegated to purple flower indicates excision of *Tgm9*, and its insertion at a new locus. Previously, we have identified a male-sterile, female-sterile mutant among the selfed progenies of a revertant plant carrying only purple flowers. Co-segregation between *Tgm9* and the sterility phenotype suggested that the mutant was generated by insertion of *Tgm9* at the *St8* locus. The transposon was localized to exon 10 of *Glyma*.*16G072300* that shows high identity to the MER3 DNA helicase involved in crossing over. Molecular analysis of fertile branches from two independent revertant plants confirmed precise excision of *Tgm9* from the *st8* allele, which restored fertility. In soybean, the gene is expressed in flower-buds, trifoliate leaves and stem. Phylogenetic analysis placed *St8* in a clade with the Arabidopsis and rice *MER3* suggesting that *St8* is most likely the orthologous *MER3* soybean gene. This study established the utility of *Tgm9* in gene identification as well as in forward and reverse genetics studies.

## Introduction

In soybean {*Glycine max* (L.) Merr.}, pigmentation in flowers and hypocotyls is regulated by five loci: *W1*, *W3*, *W4*, *Wm*, and *Wp* [1]. Mutations in the *W4* locus result in altered pigment accumulation patterns in the flowers and the hypocotyls. The *w4-m* allele is characterized by variegated flowers and green hypocotyls with purple stripes [2]. Loss of pigmentation in the *w4-m* mutants was the result of the insertion of *Tgm9*, a 20,548-bp CACTA-like transposable element, into the second intron of the dihydroflavonol-4-reductase 2 (*DFR2*) gene [3]. The mutable *w4-m* line is highly active and shows high germinal reversion frequency, about 6% per generation in *DFR2* [4]. The *Tgm9* transposable element in the *w4-m* line provides a valuable tool for identifying mutations in soybean genes. In addition, the element excises from the new insertion loci reconstituting the wild-type phenotype, an essential step for gene identification. By screening the germinal revertants carrying only purple flowers, new mutations presumably caused by insertion of *Tgm9* in new loci can be identified. Several types of mutants have been identified among progenies of germinal revertants of the *w4-m* allele; viz., root necrosis mutants [5], chlorophyll deficient mutants [6], partial female sterile mutants [7], male-sterile, female-fertile (MSFF) mutants [8] and male-sterile, female-sterile (MSFS) mutants [9–11].

Plants which exhibit the MSFS phenotype rarely produce seed and have typically been identified as synaptic mutants [12]. In higher eukaryotic organisms meiosis is a highly conserved cellular event for recombination of genes to create new genetic variation through sexual reproduction. The product of meiosis is four different haploid cells. Homologous chromosomes pair during the synapsis stage to allow crossing-over and exchange of genetic materials. There are many genes involved in the process of chromosome pairing, recombination and chromosomal separation. Mutations in these genes can lead to a lack of pairing, mis-pairing, non-disjunction, and other problems in the formation of gametes [13, 14]. Problems in gamete formation can then lead to sterility [12]. In soybean, there are many loci identified as MSFS, MSFF, or male partial sterile and female partial sterile [8, 10–12, 15].

A MSFS mutant line (ASR-10-181) was identified from the high frequency, late excision mutable category from the progeny of *w4-m* line [11]. The MSFS (*St_A06-321*) gene was mapped to the *St8* locus on MLG J [10, 11]. Among 578 F_2_ plants, 145 MSFS plants produced no seed. One exceptional MSFS F_2_ plant produced seven seeds in two 3-seeded pods and one 1-seeded pod on a single node [11]. The seven seeds produced five fertile and two sterile plants suggesting a possible reversion event in one copy of the MSFS gene before gamete formation in those three pods. However, due to segregation of the restored functional MSFS allele we observed a 3 fertile: 1 sterile ratio among the progenies. The MSFS gene was mapped to a 62 kb region on Chromosome 16; and the fertility/sterility phenotype was shown to be associated with *Tgm9* [11]. In the current study we showed that the MSFS (*St_A06-321*) gene is allelic to *St8*. We cloned the *St8* gene, which encodes MER3 DNA helicase.

## Materials and Methods

### Genetic Materials

An F_2_ population (A06-321) was generated from a cross Minsoy (PI 27890) x ASR-10-181 (A05-221). We temporarily named the sterility gene as *st_A06-321* based on the population name [11]. Fertile plants (*St_A06-321 St_A06-321* or *St_A06-321 st_A06-321*) in segregating entry (A05-221) were used as male parents and were crossed to Minsoy as a female parent. The F_1_ seeds were advanced to the F_2_ generation at the University of Puerto Rico/Iowa State University soybean nursery near Isabela, Puerto Rico. The F_2:3_ progeny rows were grown at the Bruner Farm near Ames, Iowa in 2006. Progenies of four F_2_ plants segregating for fertile and sterile plants were used in this study.

### Complementation test

In a previous investigation we mapped the MSFS gene, *st_A06-321*, to the *St8* locus [11]. To confirm if *st_A06-321* is the *St8* gene we conducted complementation test by crossing *St8 st8* x *St_A06-321 st_A06-321* and analyzed progenies for fertility.

### Genome Walking

GenomeWalker Universal kit (Clontech Laboratories, Inc., Mountain View, CA, USA) was used to determine the unknown *Tgm9*-insertion site as per the manufacturer’s instruction. Fertile and sterile bulks were made from the F_2_ population. The fertile bulk was constituted by mixing equal amounts of DNA from 10 homozygous fertile plants and the sterile bulk included a mixture of an equal amount of DNA from 10 homozygous sterile plants. DNA from fertile and sterile bulks was independently digested with four restriction enzymes *Dra*I, *Eco*RV, *Pvu*II, and *Stu*I, to generate fragments with blunt ends. After phenol: chloroform purification, digested genomic DNA samples were ligated to the GenomeWalker adaptor to generate the adaptor-ligated genomic DNA libraries. Four libraries were created for both homozygous sterile and fertile plants and were used for the first PCR along with the outer adaptor primer (AP1) and an outer transposon-specific primer (Trans R1) (Table 1). The first PCR mixture was then diluted to 100 times and used as a template for a second or “nested” PCR with the nested adaptor primer (AP2) and a nested transposon-specific primer (Trans R2) (Table 1). The bands specific to the libraries generated from sterile bulks were sequenced at the DNA facility, Iowa State University, Ames and sequences were compared with the soybean genome sequence at the Phytozome database [16] using Basic Local Alignment Search Tool (BLAST).

**Table 1:** Sequences of adaptor specific primers (AP1 and AP2), *Tgm9* specific primers (Trans R1 and Trans R2) and *Glyma*.*16G072300* specific primers (Rev1 and Rev 2).

Primer Name	Primer Description	Primer Sequence (5’ to 3’)
AP1	Outer adaptor primer	GTAATACGACTCACTATAGGGC
AP2	Nested adaptor primer	ACTATAGGGCACGCGTGGT
Trans R1	Outer transposon primer	CGTCGTGGGTGAAGAGTGGGTGAAGAGTG
Trans R2	Nested transposon primer	GCCACCCAGCGAGTTACTAAGATG
Rev1	Revertant primer 1	GGCTGAGAGAGGCTTCTTTATCTTG
Rev2	Revertant primer 2	GAGCGGTCATATTCCATATAGAGAC

### Phylogenetic Tree construction

The protein sequence for Glyma.16G072300 in soybean was found by performing a search on the Phytozome v10 database (www.phytozome.net/soybean) [16]. The Glyma.16G072300 sequence was used in a protein BLAST search using the NCBI-BLAST web service (http://blast.ncbi.nlm.nih.gov/Blast.cgi) [17] to find mutant and homologous sequences from *Glycine max*, *Oryza sativa*, *Arabidopsis thaliana*, *Zea mays*, *Sorghum bicolor*, *Triticum aestivum* and *Saccharomyces cerevisiae* (S1 Table). The protein sequences were aligned in MEGA 6.0, a phylogenetic alignment software program [18, 19] using the MUSCLE alignment [20]. The phylogenetic relationship between St8 (Glyma.16G072300), and 18 homologous proteins was then constructed using the Neighbor-Joining Method in MEGA 6.0. The evolutionary distances were computed using the Poisson Correction Method and are in units of the number of amino acid substitutions per site [21]. The analysis involved 19 protein sequences in a 10,000 replicate bootstrap test. All ambiguous positions were removed for each sequence pair. There were a total of 308 positions in the final data set.

The protein sequences for Glyma.16G072300 and the three homologs from *Oryza sativa*, *Arabidopsis thaliana* and *Saccharomyces cerevisia*e were aligned using the protein multiple sequence alignment in ClustalW (*ClustalW version 2*.*0*., 2007) (http://www.ebi.ac.uk/Tools/msa/clustalw2/) using the slow pairwise alignment and then colored to highlight conserved amino acids.

### Revertant analysis

Genomic DNA was isolated from both fertile branches (branches which have seed bearing pods) and sterile branches (branches without any pods) of two plants showing reversion from the sterile to fertile phenotype. Primers flanking the identified *Tgm9* insertion site in *St8* gene (Rev1 and Rev2) and Trans R1 were used for checking insertion of *Tgm9* in the *St8* gene (Table 1). For PCR the following conditions were used: 94°C for 2 min, 35 cycles of 94°C for 30s, 60°C for 30s and 72°C for 90s with a final extension time of 72°C for 10 min. The resulting products were checked on a 1% agarose gel. For sequencing, the PCR products were run on a 0.8% agarose gel and gel extraction was conducted using a gel extraction kit following manufacturer’s instructions (Qiagen Inc., Valencia, CA, USA). Sequencing was conducted using Rev1 and Rev2 primers (Table 1) at the DNA Facility at Iowa State University.

### RT-PCR

Tissues were harvested from flowers, flower buds, unifoliate leaves, trifoliate leaves, cotyledons and young roots of soybean plants. Tissues were frozen in liquid nitrogen and total RNA was isolated from each tissue using the Qiagen RNeasy mini kit following manufacturer’s instructions (Qiagen Inc., Valencia, CA, USA). RNA was treated with DNase to remove residual DNA contamination using RQ1 DNase (Promega, Madison, WI) following manufacturer’s instructions. cDNA was synthesized from the DNase treated RNA using MMLV reverse transcriptase following manufacturer’s instructions (Promega, Madison, WI). cDNA was diluted 5x before gene amplification. PCR cycles of 94 for 2 min, followed by 40 repeats of 94°C for 30 sec, 50°C for 30 sec and 72°C for 1 min, then followed by 72°C for 10 min. PCR products were run on a 2% agarose gel. The *Elf1B* gene, that displays constitutive expression, was included in the RT-PCR analyses to serve as a control [22]. Primers were designed from unique regions of each of the *MER3*-like gene family. Sequences for designing primers were downloaded from Phytozome (http://www.phytozome.net/soybean).

## Results

The MSFS gene, *st_A06-321*, was mapped to the *St8* locus [11]. To determine if *st_A06-321* and *St8* are allelic, complementation test was conducted by crossing *St8 st8* x *St_A06-321 st_A06-321*. Of the 45 plants generated in F_1_, 34 were fertile whereas 11 were sterile displaying segregation of 3 fertile: 1 sterile. If *St_A06-321* and *St8* were non-allelic all the F_1_ plants were expected to be fertile. However, segregation of 3 fertile: 1 sterile suggested that *St8* and *st_A06-321* are allelic. The segregation pattern was further confirmed using χ^2^ test in F_2_ generation. Of the 34 fertile F_1_ plants, 24 segregated into fertile: sterile in F_2_, confirming the expected 1 non-segregating: 2 segregating ratio with a *P* value of 0.63. Therefore, we termed *st_A06-321* as the *St8* gene.

**Fig 1:**
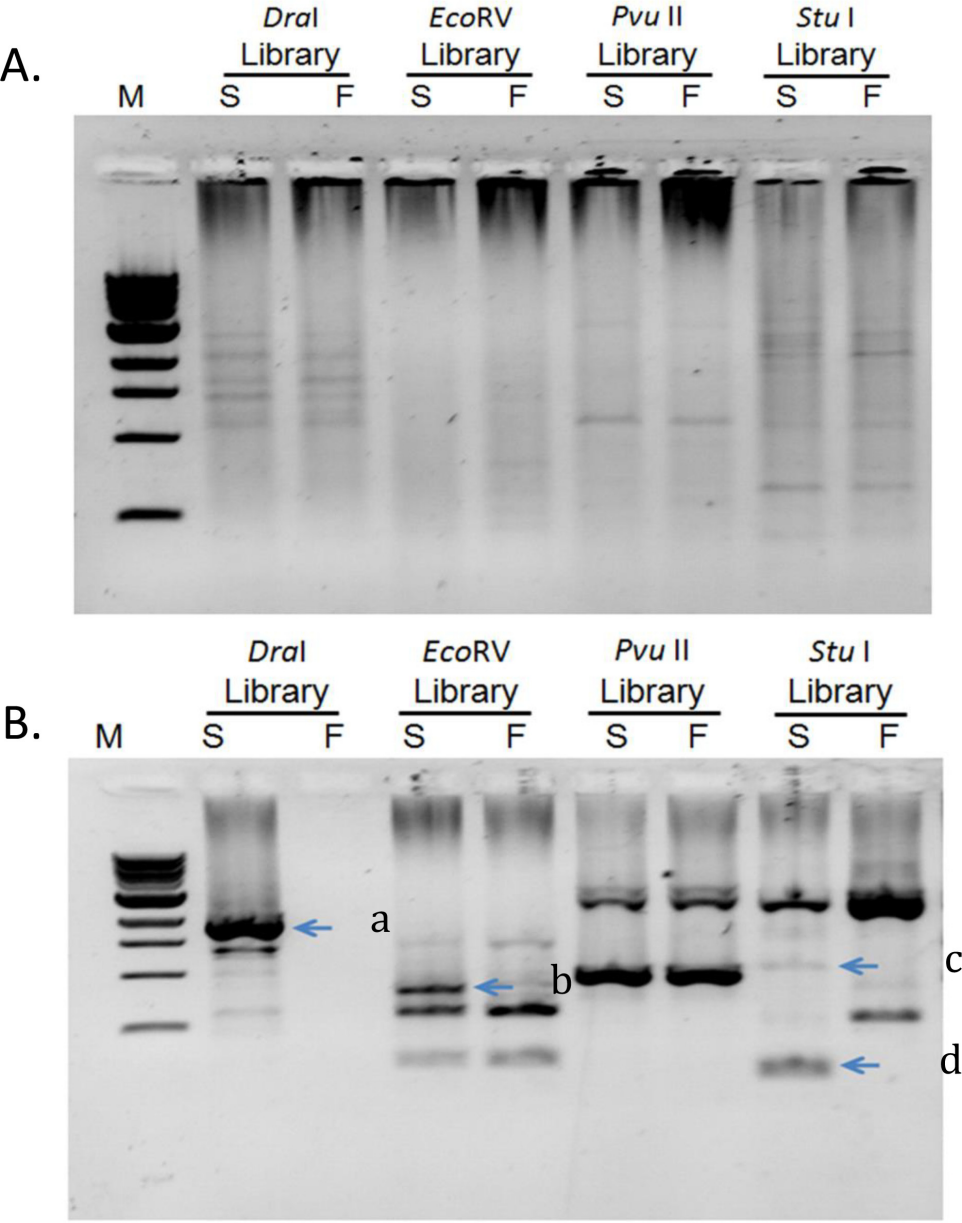
PCR amplification of the four genome walking libraries generated from the homozygous sterile bulk and the homozygous fertile bulk. **A)** Results of the first PCR (PCR1) reaction with adaptor primer (AP1) and transposon specific primer (Trans R1). **B)** Results of the second PCR (PCR2) with nested adaptor primer (AP2) and nested transposon specific primer (Trans R2). M, 200 bp DNA Ladder; S, Sterile bulk (library generated from DNA of pooled 10 homozygous sterile plants); F, Fertile Bulk (library generated from DNA of pooled 10 homozygous fertile plants). Arrows indicate the bands specific to sterile plants. a, *Dra*1-sterile-AP2; b, *Eco*RV-sterile-AP2; c, *Stu*I-sterile-AP2-1; d, *Stu*I-sterile-AP2-2.

**Fig 2:**
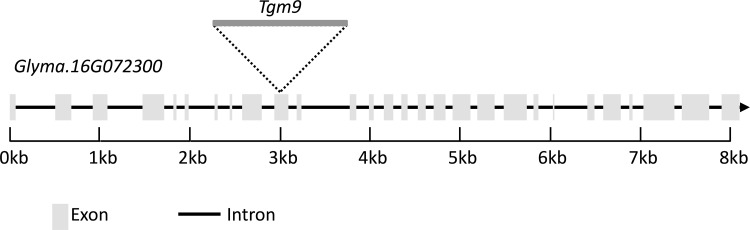
The graphical representation of *Glyma*.*16G072300*. The gene has 28 predicted exons and the transposon *Tgm9* is inserted into exon 10. The *Tgm9* insertion is not drawn to scale.

The genome walking technique was used to locate the specific insertion site of the *Tgm9* transposon in the soybean genome of the MSFS plants. Four libraries of homozygous fertile and sterile bulked samples were used for PCR amplification with the adaptor primer (AP1) and a transposon-specific primer (Trans R1) (Table 1). There were no considerable differences visible in the first round of PCR amplification between the fertile and sterile samples (Fig 1A). The first PCR product was then diluted 100 times and used in a nested PCR reaction with the adaptor primer (AP2) and the nested transposon primer (Trans R2) (Table 1). This nested PCR reaction provided bands that were unique to the sterile bulks (Fig 1B). These unique fragments were excised and used for sequencing and identifying the location of the *Tgm9* insertion. The sequences were aligned with the 5’ end of the transposon using ClustalW2 (http://www.ebi.ac.uk/Tools/msa/clustalw2/). Comparison of the sequences with the *Tgm9* sequence showed identity between the 5’ end of the *Tgm9* sequence and the four sequences (S1 Fig). The portion of the sequence which did not show identity to the *Tgm9* sequence was identical in three of the four libraries (*Dra*1-sterile-AP2, *Eco*RV-sterile-AP2, and *Stu*I-sterile-AP2-1; S1 Fig). The non-transposon sequence was used in BLAST against the soybean genome using Phytozome [16]. The sequence is identical to predicted soybean gene *Glyma*.*16G072300*. The cloned gene is located to the previously determined location on soybean chromosome 16 based on linkage mapping experiments [11]. The predicted gene *Glyma*.*16G072300* is 8,092 bp long and has 28 exons (Fig 2). Intron exon boundaries were confirmed by comparing predicted transcript sequence with RNA-seq data [23]. *Glyma*.*16G072300* encodes a 1,195 amino acid long protein carrying three prominent domains; (i) Sec63 Brl domain, (ii) DEAD/DEAH box helicase domain, and (iii) helicase conserved C-terminal domain. The *Tgm9* was inserted into the exon 10 of the gene (Fig 2).

**Fig 3:**
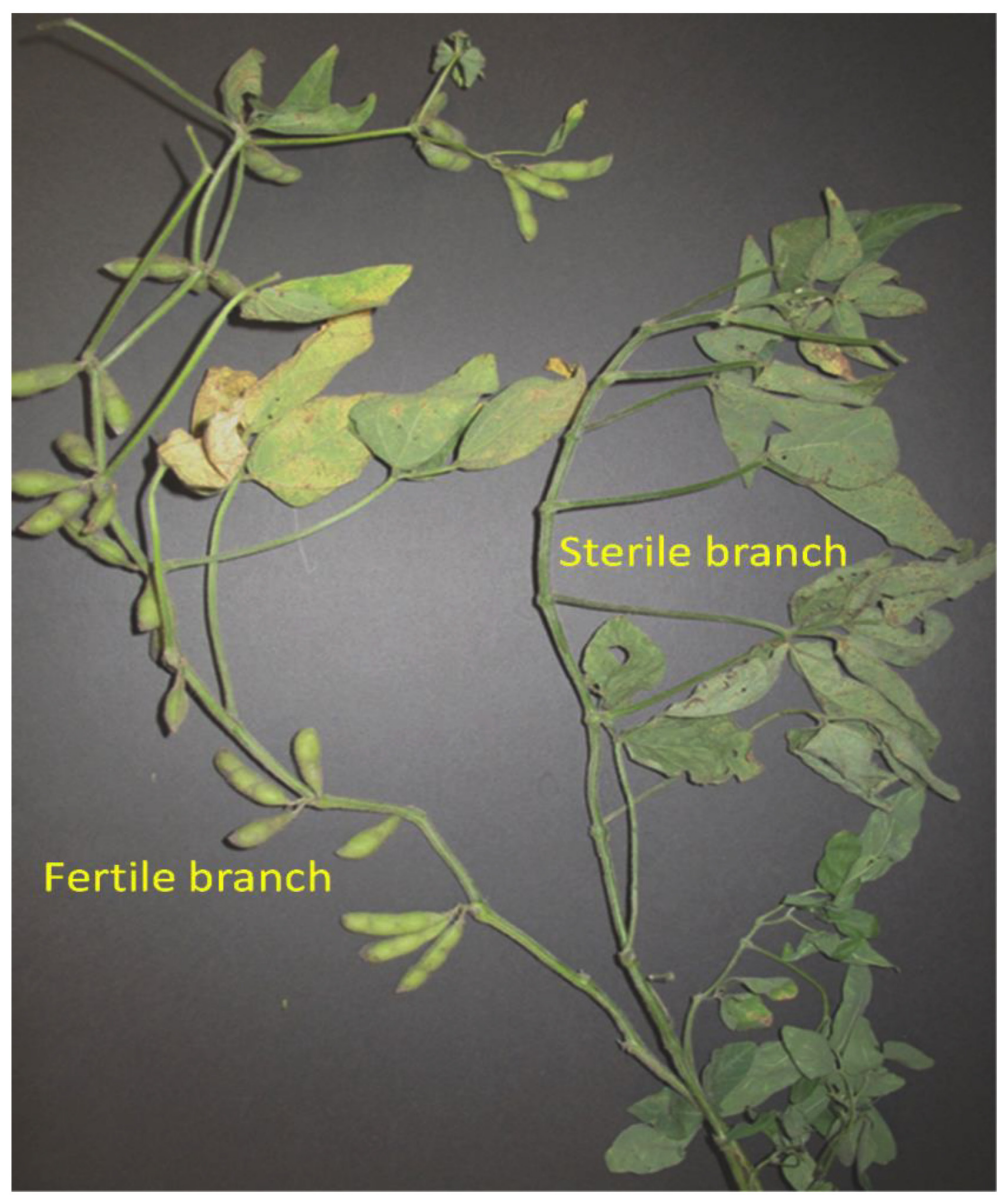
A revertant plant showing a fertile branch. The transposon excised out of the *st8* allele in the bud that resulted in the fertile branch with multiple pods bearing viable seeds. All other branches on the MSFS plant remained sterile.

**Fig 4:**
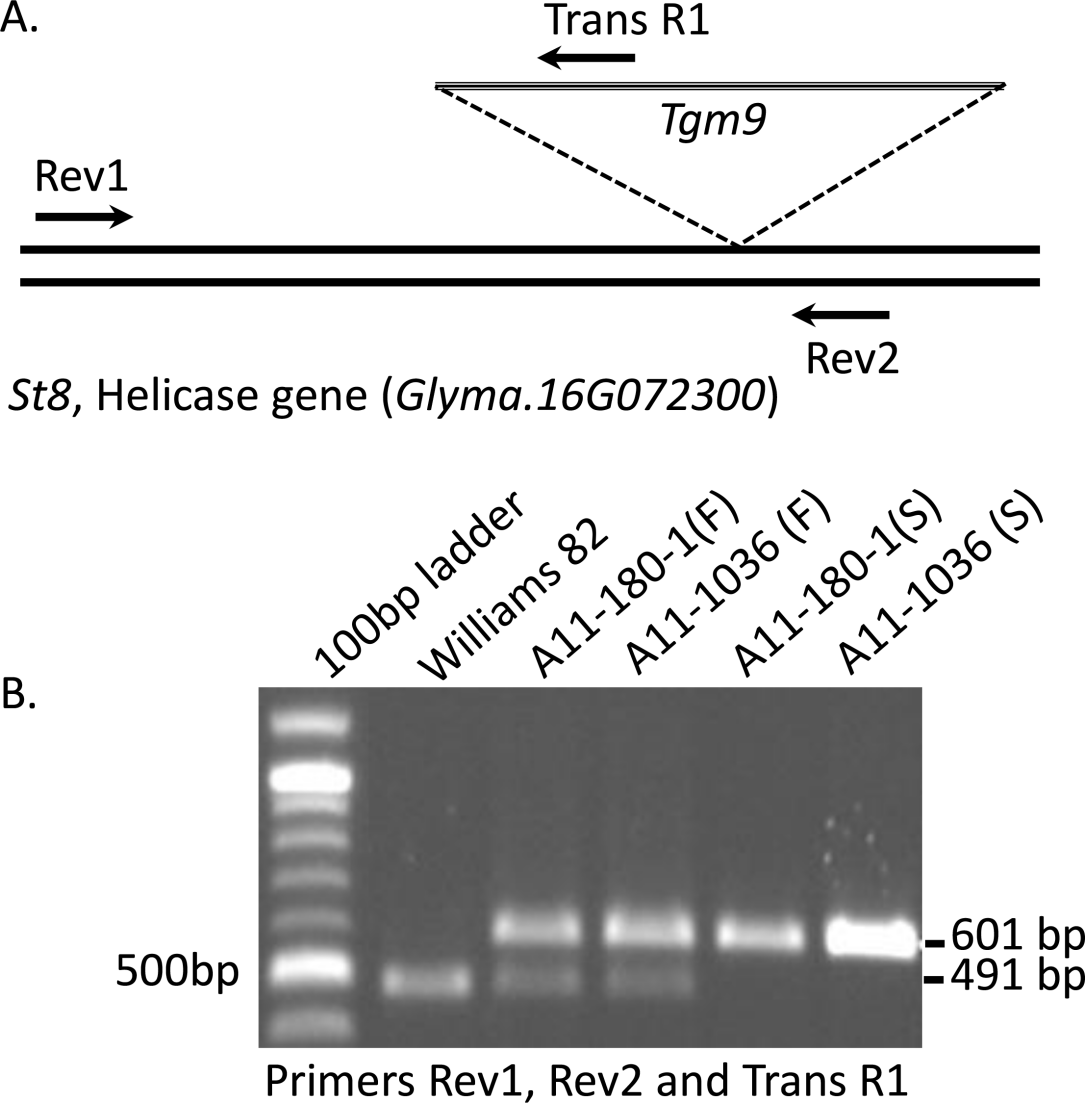
PCR analysis of two revertant plants. **A)** Graphical representation showing locations of two PCR primers (Rev1 and Rev2) flanking the insertion site in *Glyma*.*16G072300* (the *St8* gene) and a third primer (Trans R1) located in *Tgm9*. With no transposon present, the primers Rev1 and Rev2 should amplify a 491 bp fragment. With *Tgm9* present, the primers Rev1 and Trans R1 should amplify a 601 bp fragment. The *Tgm9* insertion is not drawn to scale. **B)** PCR amplification of fragments from sterile and fertile branches of two revertant plants (A11-180-1 and A11-1036) using primers Rev1, Rev2 and Trans R1. In the Williams 82 sample the transposon is not present, so only 491 bp fragment is amplified. The fertile branches of A11-180-1 and A11-1036 are heterozygous, therefore both the fragments representing absence of *Tgm9* (491 bp fragment) and presence of *Tgm9* (601 bp fragment) were amplified. The sterile branches are homozygous for the insertion of *Tgm9*, so only 601 bp fragment is amplified. F, fertile branch; S, sterile branch.

Detailed analysis of 2,429 F_2_ plants of the A06-321 population resulted in identification of 601 sterile plants. Of these, 31 produced one to three pods presumably due to precise excision of *Tgm9* from the *Glyma*.*16G072300* locus. The mutant plants carried predominantly sterile branches, but occasionally one or more branches developed fertile pods. Fig 3 shows one such revertant plant with no pod setting on the main branch, yet a fertile pod setting on a side branch. To confirm excision of the transposon in this branch with fertile pods, molecular analysis was conducted on DNA collected from fertile and sterile branches of two revertant plants. DNA from the fertile and sterile branches of the same plant was extracted separately. PCR was conducted using one forward—Rev1, and two reverse—Rev2, and Trans R1 primers at the same time (Fig 4). Only a single 601 bp DNA fragment amplified from the sterile branch suggesting that sterile branches were homozygous for *Tgm9*-insertion in *St8* (Fig 4). Two DNA fragments were amplified from the fertile branch. One band was identical to that found in the sterile branch suggesting that one *St8* copy carries *Tgm9*, while the other copy does not carry the element. The amplified 491 bp DNA fragment is presumably the wild-type copy of the *St8* gene, from which *Tgm9* was excised (Fig 4). The fertile branches were hemizygous for *Tgm9* insertion (Fig 4). Sequencing both fragments confirmed that *Tgm9* was precisely excised from the *St8* gene (S2 Fig).

**Fig 5:**
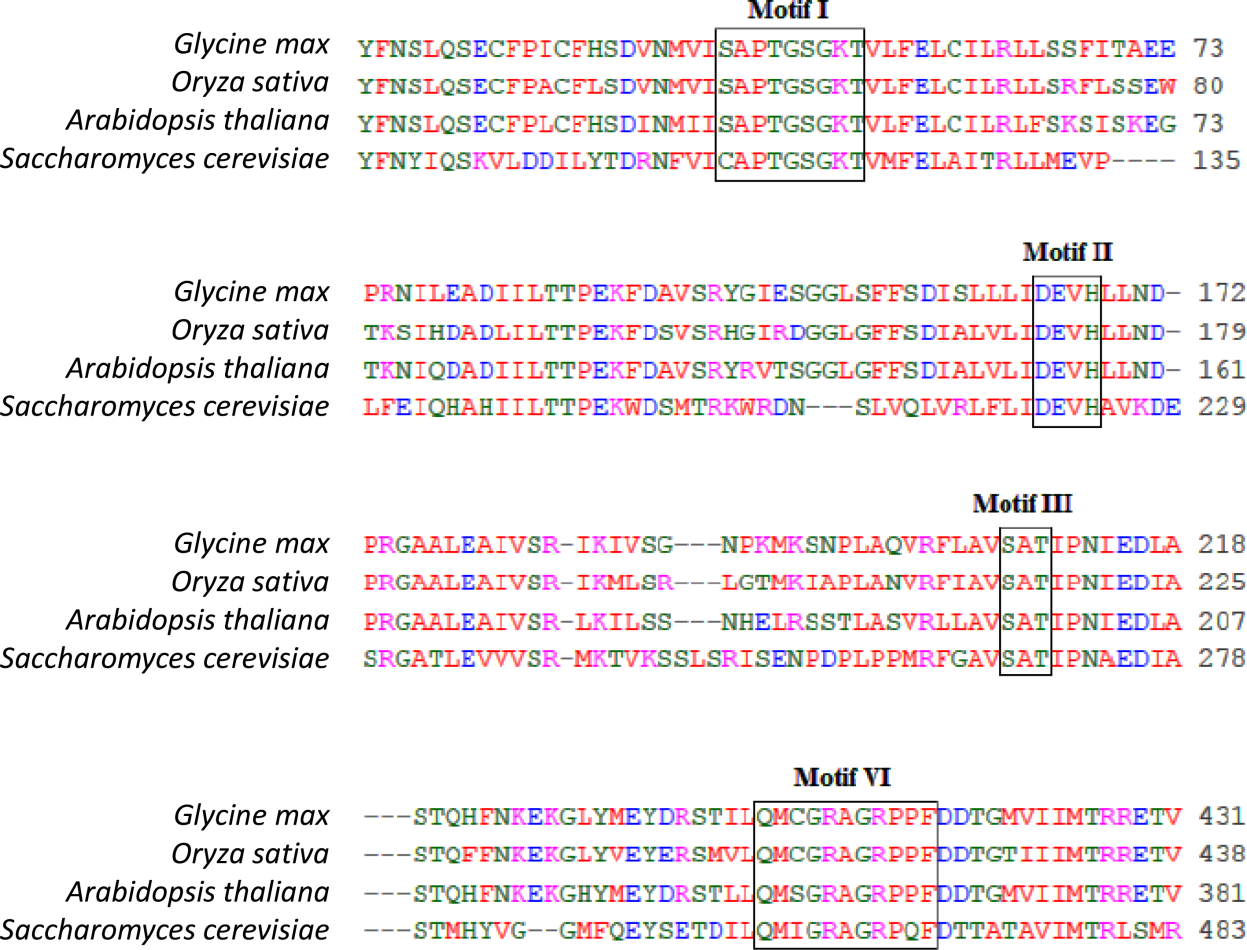
The multiple sequence alignment of soybean, rice, Arabidopsis and yeast MER3 helicases highlighting four conserved motifs commonly found in the MER3 helicases. There is 100% conservation of all the amino acid residues in four motifs among the four genes showing that the newly cloned *Glyma*.*16G072300 (St8)* gene is a MER3 DNA helicase. Only a part of the proteins is shown.

**Fig 6:**
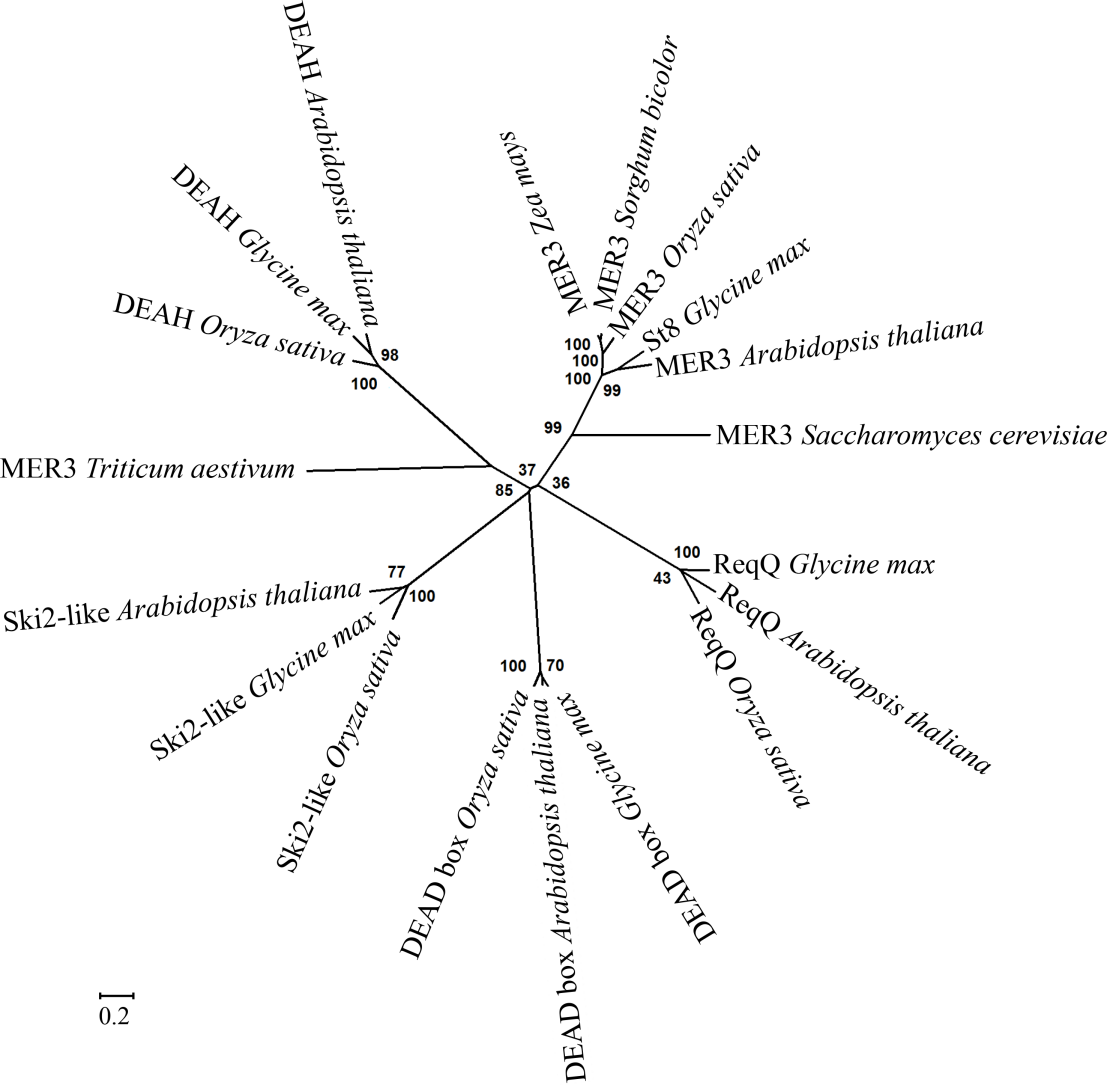
Phylogenetic tree of homologs of Glyma.16G072300 (St8). The unrooted radiated phylogenetic tree shows the relationships among the soybean helicase, St8 and homologous proteins in soybean and other taxa. The tree was generated using the Neighbor-Joining method in the MEGA6 software. Numbers on the nodes represent percent bootstrap support in a 10,000 replicate bootstrap test, and the sum of branch lengths is 9.42170767. The evolutionary distance represented by the scale bar shows 0.2 amino acid substitutions per site.

St8 showed 63% identity with over 880 amino acids of the Arabidopsis MER3 protein, which is a DEAD-box superfamily member and has been shown to be involved in recombination and cytokinesis [24–26]. It also showed 67% identity to the rice meiotic crossover 1 protein [27], and 33% identity to the yeast MER3 protein over 752 amino acids residues [26]. The high identity of the St8 protein sequence with that of the other MER3 proteins supported that the MER3 protein was the sterility gene. Alignment of St8 with the Arabidopsis, rice and yeast MER3 homologs showed conservation of the motifs that are used in characterizing MER3 proteins (Fig 5). Phylogenetic analysis of the MER3 protein family revealed that St8 was clustered with the Arabidopsis and rice MER3 proteins; and the other four soybean proteins with similarity to St8 group into four distinct clusters (Fig 6; S1 Table). This result suggests that *St8* is the only *MER3*-like soybean gene; loss of function of which resulted in the loss of fertility. Maize and sorghum MER3 homologs showed close association with soybean MER3 protein indicating high conservation of this protein in evolutionary diverse species (Fig 6).

**Fig 7:**
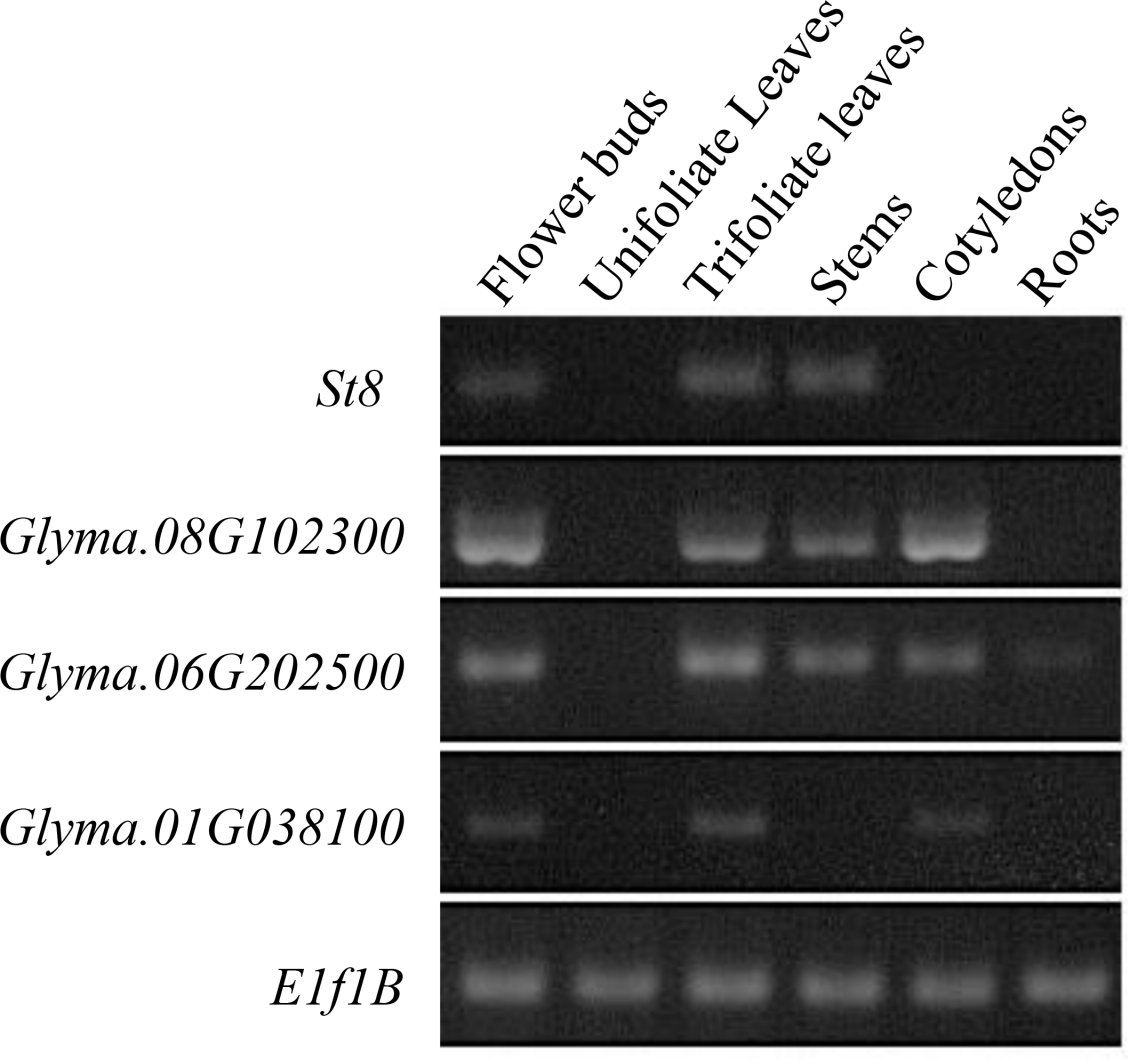
Semi-quantitative RT-PCR showing expression of *St8* in different soybean tissues. *Glyma*.*16G072300* showed an expression pattern distinct from the other DEAH/D-box helicase genes (*Glyma*.*08g102300*, *Glyma*.*06g202500*, and *Glyma*.*01g038100*) in soybean. The *Elf1B* gene was used to confirm equal loading of RNA for each sample.

To further characterize *St8*, the expression profiles of all five DEAH/D domain containing soybean genes were investigated. No transcripts were detected for *Glyma*.*08g174300* in the tissues taken for this study (data not presented). The expression profiles of *St8* were distinct from that of the other three DEAH/D domain containing genes. *St8* is expressed in flower buds, trifoliate leaves, and stems (Fig 7). All other DEAH/D domain containing genes were expressed in cotyledons. None of the DEAH/D genes were expressed in unifoliate leaves. Expression pattern suggested that although multiple DNA helicase-like genes are expressed in flower buds and trifoliate leaves, they show different expression pattern in other organs. Publicly available transcriptomic data also supported that *st8* and it homologous genes are highly expressed in flower buds, inflorescence or other reproductive organs (S1 Fig) [23, 28–30].

## Discussion

In this investigation we have shown the utility of *Tgm9* in isolating a MSFS gene, *St8*, in soybean. *Tgm9* is an active CACTA-type transposable element highly similar to Tgmt* which was isolated from the soybean t* allele [3, 31]. *Tgm9* is a low copy transposable element [3, 32, 33]. *Tgm9* is known to produce two distinct transposases through alternate splicing and may have similar excision mechanism as *En/Spm* [3, 34]. It occasionally fractures during excision resulting in stable mutations [17]. In the *w4-m* line, the element is located in intron 2 of the *DFR2* gene involved in anthocyanin pigment biosynthesis in petals and hypocotyls [3]. Due to the presence of the element in this gene, pigment biosynthesis is interrupted and white flowers are produced in this mutant line. *Tgm9* is a highly active element, and upon precise or imprecise excision from the flower color gene, purple flower color is developed either in petal sectors (excision of the element in the somatic tissues; somatic excision) or in entire petals (excision of the element in progenitor cells that led to flower development) [3]. When the entire flower is purple, some of the progenies developed from this flower bear only purple flowers; and these events are in germinal cells and are called germinal revertants. From the progeny of a germinal revertant carrying purple flowers, a MSFS mutant was identified and the mutant fertility gene was mapped to the *St8* locus on molecular linkage group J [11]. The sterility gene was temporarily named as *st_A06-321*.

We conducted a complementation test to confirm that *st_A06-321* and *St8* are allelic. The mutant *st_A06-321* allele was presumably generated from insertion of *Tgm9* excised from the *W4* locus. Earlier we have shown co-segregation of the mutant *st_A06-321* allele with a *Tgm9* insertion [11]. We have cloned the *St8* gene using *Tgm9*. This is the first fertility gene cloned in soybean. Previously, we reported candidates for the fertility gene, *St8* based on fine mapping of the *St8* region containing a MSFS gene [11]. Sequencing of the *Tgm9*-insertion site in the MSFS mutant established that one of the five candidate genes, *Glyma*.*16G072300*, that encodes MER3 DNA helicase is most likely the *St8* gene, which regulates the male and female fertility in soybean.

The MSFS mutant plants are completely sterile. Occasionally, a branch of some sterile plants developed pods with seeds. These revertant branches provided us evidence to establish that *Glyma*.*16G072300* is the *St8* gene. Molecular analysis of the revertant branches established that precise excision of *Tgm9* from *Glyma*.*16G072300* of the MSFS mutant led to recovery of the fertility phenotype (Fig 3; Fig 4; S2 Fig). Fertile branches were found to contain one functional copy of the *Glyma*.*16G072300* gene. This confirmed that *Glyma*.*16G072300* is the *St8* gene that encodes a MER3 DNA helicase.

The protein encoded by *Glyma*.*16G072300* shows 63% and 67% identities to the Arabidopsis Rock-N-Rollers (RCK) MER3 DNA helicase and the rice meiotic crossover 1, respectively [27, 35]. The *MER3* gene is critical for meiotic crossing over [35]. The *mer3* mutants display defects in bivalent formation during chromosome pairing and flaws in crossover formation, reduced chiasma formation, interference insensitivity, polyad anthers and reduced viability of the pollen grains resulting in reduced fertility [27, 35, 36]. RAD51 is known to be involved in synaptonemal complex formation and recombination [37]. MER3 plays important role in unwinding of double stranded DNA and may possibly be involved in bringing RAD51 onto recombination nodule [38]. The *MER3* gene appears to be conserved in eukaryotes, across kingdoms; and appears to be an essential part of the meiotic process. Soybean contains five DEAH/D domain containing genes, four of which including *St8* are expressed in flower buds (Fig 7). However, mutation in *St8* alone results in sterility in soybean. This observation suggests that the other four DEAH/D domain-containing *Mer*-like genes are not involved in meiosis.

Meiosis requires multiple genes involved in processes such as correct chromosome pairing, crossing over and separation of chromatids. Studying mutations in genes that play key roles in meiosis will provide a better understanding of the mechanisms that are crucial for such important genetic events. Mutations that affect the meiotic process often lead to sterility. Several meiotic mutations have been identified in soybean [39]. Manual emasculation of female plants for the production of large quantities of hybrid soybean seeds can be labor intensive and tedious. Male-sterile mutants are considered to be a powerful tool in hybrid breeding programs. Understanding the network of genes involved in male and female fertility may provide an entry point for understanding and manipulating reproductive biology of soybean for hybrid breeding.

This study established the utility of the *w4-m* line in cloning a gene responsible for a sterile phenotype in soybean. Several different types of mutants such as chlorophyll deficiency/malate dehydrogenase nulls, root necrosis, female partial sterility, complete male and female sterility, and flower color/distribution pattern plants have appeared in the progenies of the *w4-m* line [6]. Mutants with easily detectable phenotypes are ideal for the molecular characterizations and gaining insight into the genes responsible for these traits in soybean. The ease of detecting transposon movement in the *w4-m* line, and localization via genome walking, make the *w4-m* system an ideal gene characterization tool for soybean. In addition, progenies of the mutable line with restored purple flower color can be screened to identify the insertion sites of the transposable element and can be used to characterize thousands of soybean genes. Most importantly, detection of the wild-type phenotypes among the mutants following excision of the element from the mutant locus eliminates the needs of transformations step for gene identification. One can easily select stable mutations from a desirable *Tgm9*-mutant because the element is large and highly fragile, and occasionally generates stable mutations during excision because of its failure to excise precisely [3]. The *Tgm9*-induced stable mutants are highly suitable for breeding programs, facilitating cultivar development without transgenic technology unhindered by the lengthy and expensive regulation process required for transgenic plants.

## Supporting Information

S1 Fig Graphical representation of sequence comparisons of *Tgm9* and four fragment bands specific to the sterile bulk. One end of all the sequences matches with transposon sequence (black bar). Gray bar represents sequence flanking *Tgm9* insertion site. Top line represents total length of sequences in base pairs.
(PPTX)

S2 Fig Alignment of the *Glyma*.*16G072300* with the sequences from sterile and fertile branches of the two revertant plants (A11-180-1 and A11-1036). Fragment from sterile branches were amplified using Rev1 and Trans R1 and fertile branches were amplified using Rev1 and Rev2. The place where the sequence ends on the sterile branches is where *Tgm9* insertion site (marked by a box) is present. Comparison of sequences from the fertile branches and *Glyma*.*16G072300* shows no footprint left by *Tgm9* in the fertile branches of the revertant plants.
(PPTX)

S3 Fig Expression analyses of soybean *st8* and its homologs in Arabidopsis, rice and maize. A) RNA-seq analysis of *st8* (*Glyma*.*16G07230)* in different soybean tissues. B) RNA-seq analysis of MER3 homolog, *AT3G27730*, in different Arabidopsis tissues. C) RNA-seq analysis of MER3 homolog, *LOC_OS02G40450*, in different rice tissues. D) RNA-seq analysis of MER3 homolog, *GRMZM2G346278*, in different maize tissues. FPKM, Fragments Per Kilobase of transcript per Million mapped reads [23, 28–30].
(PPTX)

S1 Table The protein sequences *Glycine max* St8 MER3 and 18 homologous proteins used for phylogenetic analysis. (PPTX)

## Abbreviations

SSR: simple sequence repeat
PCR: polymerase chain reaction
BSA: bulked segregant analysis
cM: centiMorgan
MLG: molecular linkage group
MSFS: male-sterile, female-sterile
MSFF: male-sterile, female-fertile
