# Supplementary material for: Transposon Tagging of a Male-Sterility, Female-Sterility Gene, St8, Revealed that the Meiotic MER3 DNA Helicase Activity Is Essential for Fertility in Soybean
Source: PLoS One. 2016 Mar 1;11(3):e0150482. doi: 10.1371/journal.pone.0150482 (PMC4773125; doi:10.1371/journal.pone.0150482)
Supplement: S1 Fig — One end of all the sequences matches with transposon sequence (black bar). Gray bar represents sequence flanking Tgm9 insertion site. Top line represents total length of sequences in base pairs. (PPTX) [file pone.0150482.s001.pptx]

## Slide 1
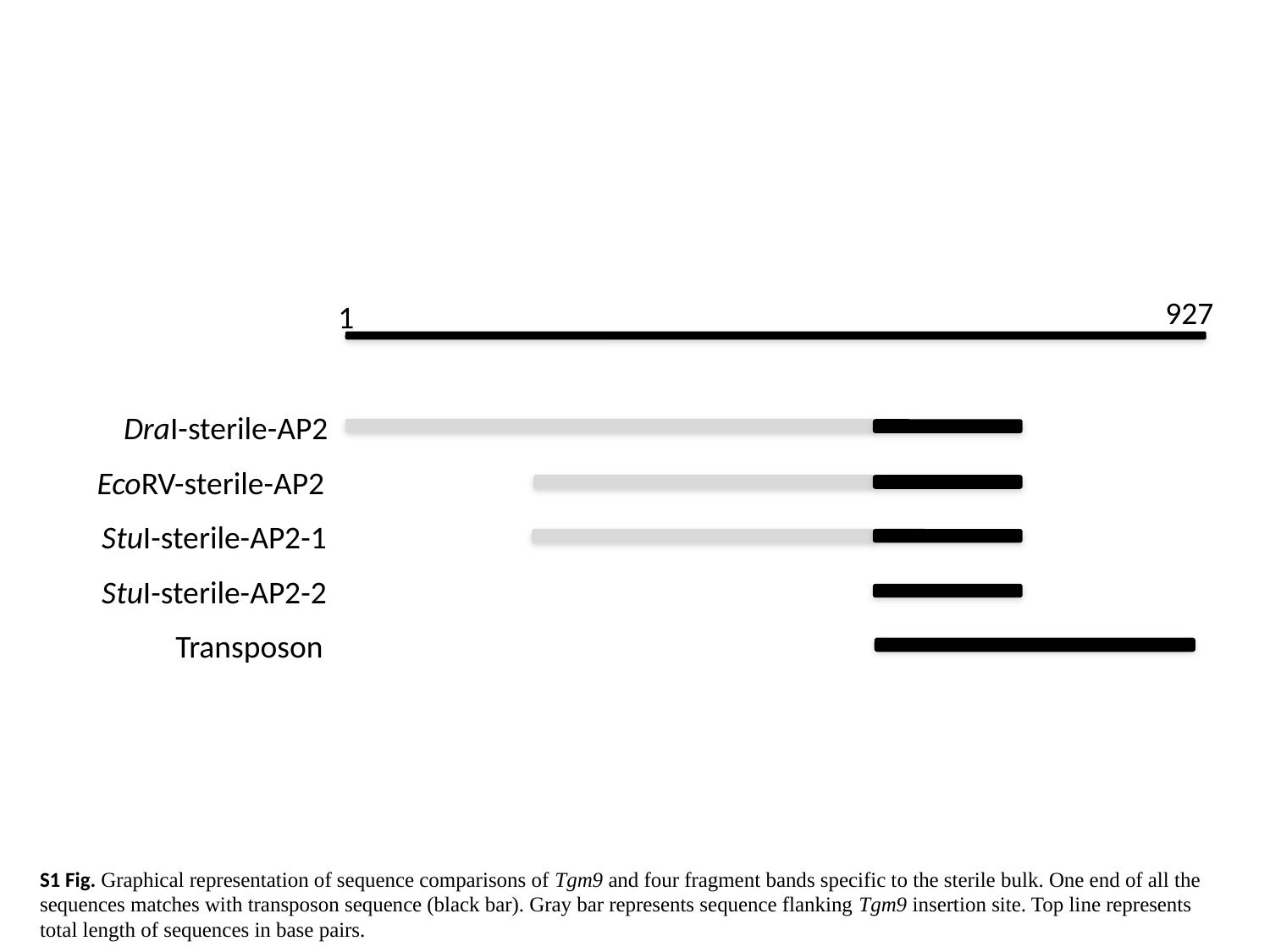

927
1
DraI-sterile-AP2
EcoRV-sterile-AP2
StuI-sterile-AP2-1
StuI-sterile-AP2-2
Transposon
S1 Fig. Graphical representation of sequence comparisons of Tgm9 and four fragment bands specific to the sterile bulk. One end of all the sequences matches with transposon sequence (black bar). Gray bar represents sequence flanking Tgm9 insertion site. Top line represents total length of sequences in base pairs.
