# Supplementary material for: Transposon Tagging of a Male-Sterility, Female-Sterility Gene, St8, Revealed that the Meiotic MER3 DNA Helicase Activity Is Essential for Fertility in Soybean
Source: PLoS One. 2016 Mar 1;11(3):e0150482. doi: 10.1371/journal.pone.0150482 (PMC4773125; doi:10.1371/journal.pone.0150482)
Supplement: S2 Fig — Fragment from sterile branches were amplified using Rev1 and Trans R1 and fertile branches were amplified using Rev1 and Rev2. The place where the sequence ends on the sterile branches is where Tgm9 insertion site (marked by a box) is present. Comparison of sequences from the fertile branches and Glyma.16G072300 shows no footprint left by Tgm9 in the fertile branches of the revertant plants. (PPTX) [file pone.0150482.s002.pptx]

## Slide 1
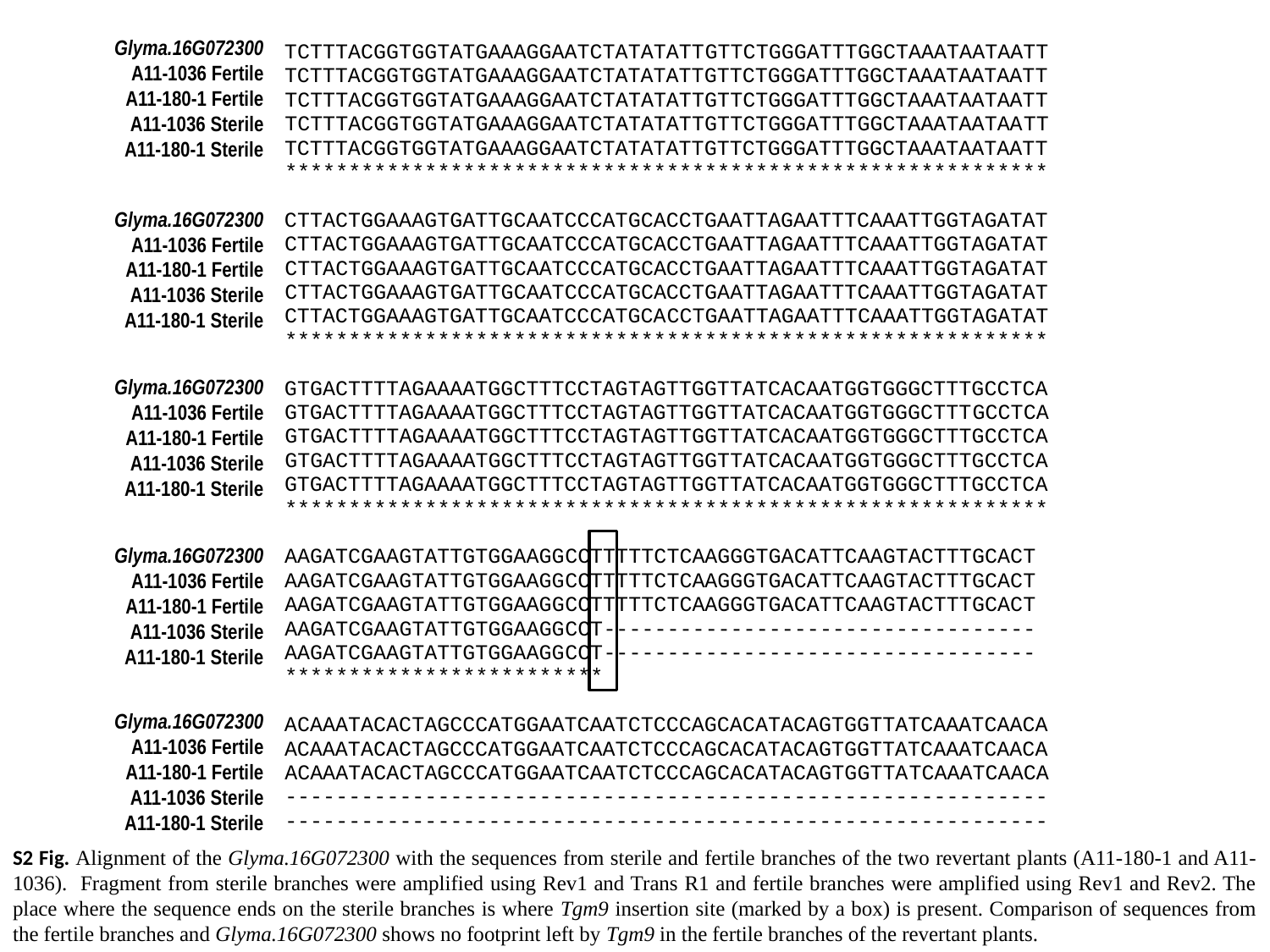

Glyma.16G072300
A11-1036 Fertile
A11-180-1 Fertile
A11-1036 Sterile
A11-180-1 Sterile
Glyma.16G072300
A11-1036 Fertile
A11-180-1 Fertile
A11-1036 Sterile
A11-180-1 Sterile
Glyma.16G072300
A11-1036 Fertile
A11-180-1 Fertile
A11-1036 Sterile
A11-180-1 Sterile
Glyma.16G072300
A11-1036 Fertile
A11-180-1 Fertile
A11-1036 Sterile
A11-180-1 Sterile
Glyma.16G072300
A11-1036 Fertile
A11-180-1 Fertile
A11-1036 Sterile
A11-180-1 Sterile
S2 Fig. Alignment of the Glyma.16G072300 with the sequences from sterile and fertile branches of the two revertant plants (A11-180-1 and A11-1036). Fragment from sterile branches were amplified using Rev1 and Trans R1 and fertile branches were amplified using Rev1 and Rev2. The place where the sequence ends on the sterile branches is where Tgm9 insertion site (marked by a box) is present. Comparison of sequences from the fertile branches and Glyma.16G072300 shows no footprint left by Tgm9 in the fertile branches of the revertant plants.
