# Supplementary material for: Transposon Tagging of a Male-Sterility, Female-Sterility Gene, St8, Revealed that the Meiotic MER3 DNA Helicase Activity Is Essential for Fertility in Soybean
Source: PLoS One. 2016 Mar 1;11(3):e0150482. doi: 10.1371/journal.pone.0150482 (PMC4773125; doi:10.1371/journal.pone.0150482)
Supplement: S3 Fig — A) RNA-seq analysis of st8 (Glyma.16G07230) in different soybean tissues. B) RNA-seq analysis of MER3 homolog, AT3G27730, in different Arabidopsis tissues. C) RNA-seq analysis of MER3 homolog, LOC_OS02G40450, in different rice tissues. D) RNA-seq analysis of MER3 homolog, GRMZM2G346278, in different maize tissues. FPKM, Fragments Per Kilobase of transcript per Million mapped reads [23, 28–30]. (PPTX) [file pone.0150482.s003.pptx]

## Slide 1
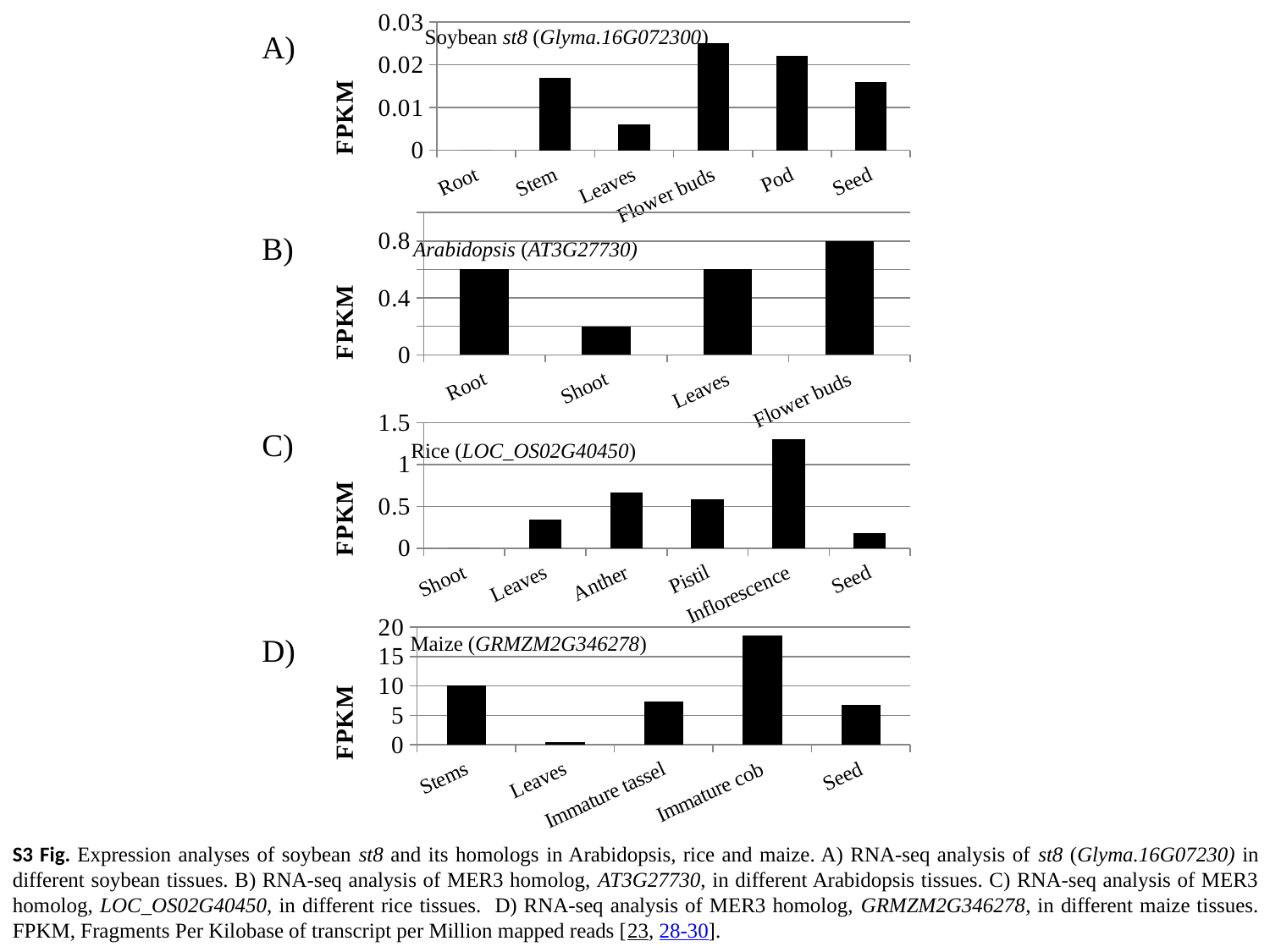

### Chart
| Category | FPKM |
|---|---|
| Root | 0.0 |
| Stem | 0.017 |
| Leaves | 0.006 |
| Flower buds | 0.025 |
| Pod | 0.022 |
| Seed | 0.016 |Soybean st8 (Glyma.16G072300)
A)
### Chart
| Category | FPKM |
|---|---|
| Root | 0.6 |
| Shoot | 0.2 |
| Leaves | 0.6 |
| Flower buds | 0.8 |B)
Arabidopsis (AT3G27730)
### Chart
| Category | |
|---|---|
| Shoot | 0.0 |
| Leaves | 0.340065 |
| Anther | 0.66562 |
| Pistil | 0.580464 |
| Inflorescence | 1.30106 |
| Seed | 0.184152 |C)
Rice (LOC_OS02G40450)
### Chart
| Category | FPKM |
|---|---|
| Stems | 10.1 |
| Leaves | 0.4 |
| Immature tassel | 7.3 |
| Immature cob | 18.5 |
| Seed | 6.8 |Maize (GRMZM2G346278)
D)
S3 Fig. Expression analyses of soybean st8 and its homologs in Arabidopsis, rice and maize. A) RNA-seq analysis of st8 (Glyma.16G07230) in different soybean tissues. B) RNA-seq analysis of MER3 homolog, AT3G27730, in different Arabidopsis tissues. C) RNA-seq analysis of MER3 homolog, LOC_OS02G40450, in different rice tissues. D) RNA-seq analysis of MER3 homolog, GRMZM2G346278, in different maize tissues. FPKM, Fragments Per Kilobase of transcript per Million mapped reads [23, 28-30].
