# Supplementary material for: Transposon Tagging of a Male-Sterility, Female-Sterility Gene, St8, Revealed that the Meiotic MER3 DNA Helicase Activity Is Essential for Fertility in Soybean
Source: PLoS One. 2016 Mar 1;11(3):e0150482. doi: 10.1371/journal.pone.0150482 (PMC4773125; doi:10.1371/journal.pone.0150482)
Supplement: S1 Table — (PPTX) [file pone.0150482.s004.pptx]

## Slide 1
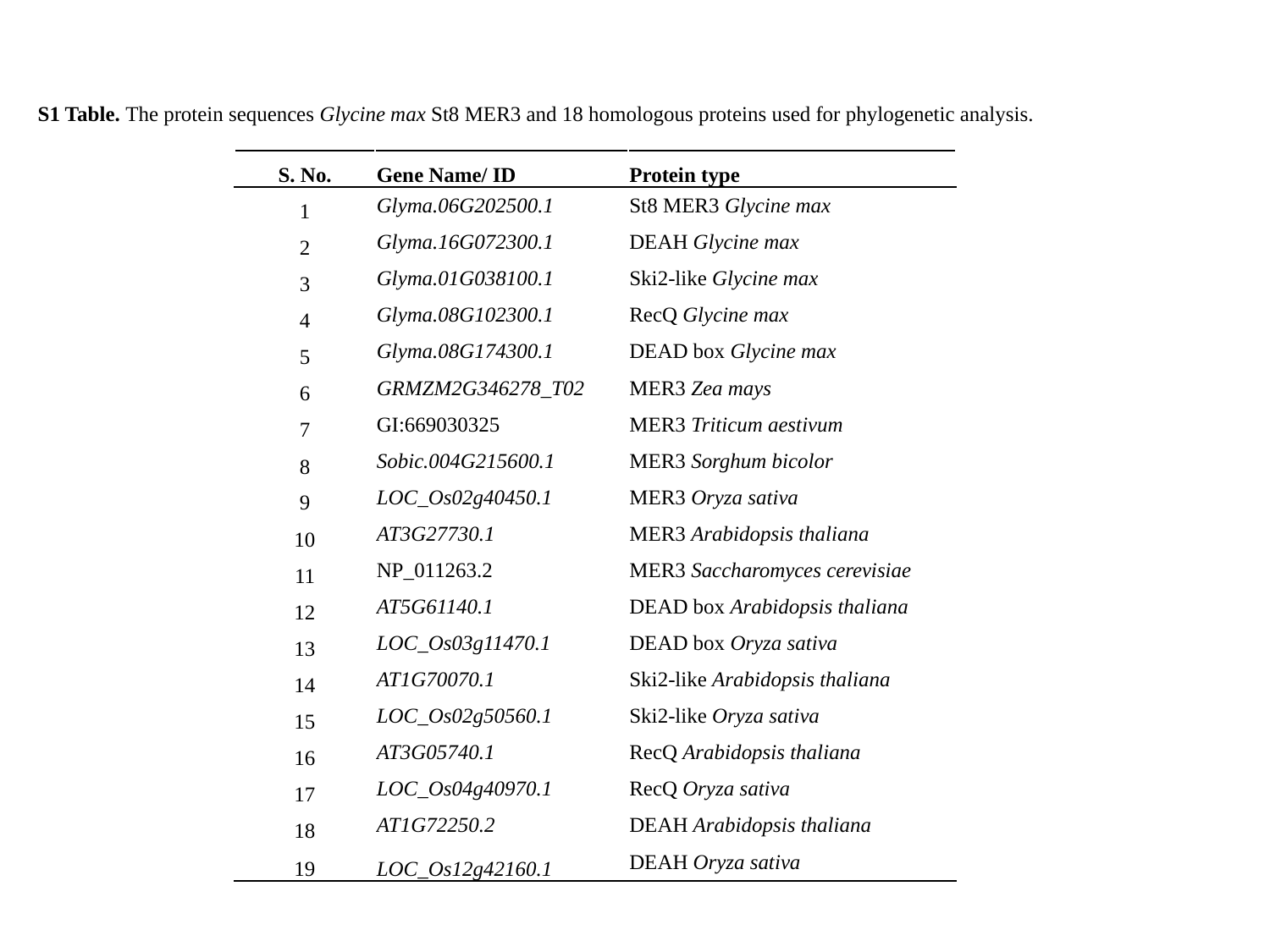

S1 Table. The protein sequences Glycine max St8 MER3 and 18 homologous proteins used for phylogenetic analysis.
| S. No. | Gene Name/ ID | Protein type |
| --- | --- | --- |
| 1 | Glyma.06G202500.1 | St8 MER3 Glycine max |
| 2 | Glyma.16G072300.1 | DEAH Glycine max |
| 3 | Glyma.01G038100.1 | Ski2-like Glycine max |
| 4 | Glyma.08G102300.1 | RecQ Glycine max |
| 5 | Glyma.08G174300.1 | DEAD box Glycine max |
| 6 | GRMZM2G346278\_T02 | MER3 Zea mays |
| 7 | GI:669030325 | MER3 Triticum aestivum |
| 8 | Sobic.004G215600.1 | MER3 Sorghum bicolor |
| 9 | LOC\_Os02g40450.1 | MER3 Oryza sativa |
| 10 | AT3G27730.1 | MER3 Arabidopsis thaliana |
| 11 | NP\_011263.2 | MER3 Saccharomyces cerevisiae |
| 12 | AT5G61140.1 | DEAD box Arabidopsis thaliana |
| 13 | LOC\_Os03g11470.1 | DEAD box Oryza sativa |
| 14 | AT1G70070.1 | Ski2-like Arabidopsis thaliana |
| 15 | LOC\_Os02g50560.1 | Ski2-like Oryza sativa |
| 16 | AT3G05740.1 | RecQ Arabidopsis thaliana |
| 17 | LOC\_Os04g40970.1 | RecQ Oryza sativa |
| 18 | AT1G72250.2 | DEAH Arabidopsis thaliana |
| 19 | LOC\_Os12g42160.1 | DEAH Oryza sativa |
